# Eye-Based Recognition of User Traits and States—A Systematic State-of-the-Art Review

**DOI:** 10.3390/jemr18020008

**Published:** 2025-04-01

**Authors:** Moritz Langner, Peyman Toreini, Alexander Maedche

**Affiliations:** Institute for Information Systems (WIN), Department of Economics and Management, Karlsruhe Institute of Technology (KIT), Kaiserstraße 89-93, 76133 Karlsruhe, Germanyalexander.maedche@kit.edu (A.M.)

**Keywords:** eye tracking, user modeling, cognitive state, affective state, trait, personality, machine learning, deep learning

## Abstract

Eye-tracking technology provides high-resolution information about a user’s visual behavior and interests. Combined with advances in machine learning, it has become possible to recognize user traits and states using eye-tracking data. Despite increasing research interest, a comprehensive systematic review of eye-based recognition approaches has been lacking. This study aimed to fill this gap by systematically reviewing and synthesizing the existing literature on the machine-learning-based recognition of user traits and states using eye-tracking data following PRISMA 2020 guidelines. The inclusion criteria focused on studies that applied eye-tracking data to recognize user traits and states with machine learning or deep learning approaches. Searches were performed in the ACM Digital Library and IEEE Xplore and the found studies were assessed for the risk of bias using standard methodological criteria. The data synthesis included a conceptual framework that covered the task, context, technology and data processing, and recognition targets. A total of 90 studies were included that encompassed a variety of tasks (e.g., visual, driving, learning) and contexts (e.g., computer screen, simulator, wild). The recognition targets included cognitive and affective states (e.g., emotions, cognitive workload) and user traits (e.g., personality, working memory). A set of various machine learning techniques, such as Support Vector Machines (SVMs), Random Forests, and deep learning models were applied to recognize user states and traits. This review identified state-of-the-art approaches and gaps, which highlighted the need for building up best practices, larger-scale datasets, and diversifying tasks and contexts. Future research should focus on improving the ecological validity, multi-modal approaches for robust user modeling, and developing gaze-adaptive systems.

## 1. Introduction

In today’s world, we are surrounded by a vast array of digital technologies that help us perform everyday tasks, such as working, learning, shopping, and communicating. Interactions with these digital technologies follow well-established paradigms. Mice, keyboards, and touchscreens are the most common input modalities of today’s digital technologies and allow for efficient and effective interaction. In many cases, users know what they want to accomplish with the PC, but the input modalities provide limited insight into these internal user processes, creating an information asymmetry, where systems are unaware of users’ intentions, frustrations, or engagement [1]. Currently, the vast amount of wearables and biosignal sensors allow for counteracting this challenge by providing digital technologies with deeper insights into the traits and states of users [2,3,4]. While user traits are characteristics of a person that persist over time (e.g., personality traits), user states (e.g., emotional or cognitive) depend on the characteristics of the user, the situation, and the interaction between the user and the situation [5]. Thus, user states vary over time as the situation changes and are context dependent [5]. By providing further information about the user state or trait, interactive systems have the ability to make the interaction between users and computers symmetrical and increase the effectiveness and efficiency of human–computer interaction [1].

Biosignals offer a powerful means of sensing user states and traits, enabling a deeper understanding of user needs and fostering more balanced interactions with computers. These signals, produced autonomously by living organisms, can be measured continuously using sensors such as electroencephalogram (EEG), electrocardiogram (ECG), electrodermal activity (EDA) sensors, or eye tracking during computer usage [3]. Eye tracking is a technology that provides access to a variety of user information from the eyes, such as eye movements, pupil size, blinks, and head distance [6]. Utilizing this information and finding correlations between user states, such as the cognitive workload, emotions, mind wandering, or traits like personality traits, has been gaining growing interest in research in recent years [7,8,9,10]. These insights can be used to develop eye-based models for recognizing and interpreting user states and traits. Advancements in machine learning have further expanded the potential for connecting eye-tracking data with user traits and states. This is underscored by recent reviews on the eye-based recognition of cognitive and affective states [11] and personality traits and cognitive abilities [12]. These studies provide initial insights into the feasibility of machine learning models for eye-based user recognition and highlight the eye-related features used. However, they do not adopt a systematic approach, leaving a gap in understanding, where specific machine learning and deep learning algorithms have been applied to model particular user traits or states. A comprehensive overview of these methods would be a valuable contribution for the advancement of eye-based user modeling. Furthermore, Steichen [12] emphasizes the need for further research that focuses on deep learning approaches and collecting data outside controlled laboratory settings. Skaramagkas et al. [11] also highlighted the potential of combining eye-tracking data with other biosignals, such as EEG, ECG, and EDA, to enable a multi-modal recognition approach. Finally, studies on eye-based user modeling is scattered across several disciplines, e.g., HCI, ML and deep learning, autonomous driving, and psychology. Thus, we argue that there is a research gap for a comprehensive overview of the current literature on eye-based user trait and state recognition across disciplines, with a focus on ML and deep learning, multi-modality, and diverse contexts to further advance the development of future eye-based user models. This led to the following research question:

RQ: What is the state-of-the-art of eye-based user trait and state recognition using ML approaches and what are the future research directions?

We conducted a systematic literature review (SLR) and developed a conceptual framework for eye-based user state and trait recognition. We found and reviewed 90 articles on the dimensions of task, context, technology and data processing, and recognition target. Based on this analysis, we derived future research directions to guide research on eye-based user trait and state recognition. We contribute a comprehensive overview of which user traits and states have been investigated and recognized using eye-tracking data, and which ML-based modeling approaches and eye-based features have been used so far. This will support researchers and practitioners in the development of future eye-based user models. Moreover, we contribute to the development of gaze-adaptive systems. Following the paradigm of biosignal-adaptive systems by Schultz and Maedche [3], gaze-adaptive systems use biosignal information from the user’s eyes by continuously recording and interpreting them to model user traits and states, and ultimately to adapt the system to the user’s current needs. Adaptive systems were researched in various disciplines, such as HCI, NeuroIS, or neuroscience [1,13,14,15], but their development still remains a challenge [3]. The first gaze-adaptive systems were developed by Hutt et al. [9] and Qi et al. [16] and show the potential of adaptive systems to support mind-wandering during learning and to help readers improve their reading strategy. Therefore, this review lays the foundation for developing the necessary user models to enable eye-based user trait and state recognition.

## 2. Foundations

Eye tracking is an established technology for recording the eye movements and pupil sizes of people [6]. Modern eye trackers rely on the video-based infrared pupil–cornea reflection technology, which creates a reflection on the cornea of the eye that moves in relation to the pupil center depending on were the user is looking [17,18]. Through a calibration process, the eye tracker can calculate the point of gaze on a screen and the gaze direction. Typically, eye-tracking devices are divided into two classes: remote eye trackers and head-mounted eye trackers [6,17,19]. Remote eye trackers (often referred to as table-mounted, screen-based, or desktop-based eye trackers) are positioned at a distance of 50–90 cm and are attached to or underneath a screen. Thus, they are not in physical contact with the user. Remote eye trackers typically provide a stream of information for the points of gaze (x- and y-coordinates on a screen) for the left and right eyes, gaze origins (x-, y-, and z-coordinates) for the left and right eyes, and pupil sizes of the left and right eyes [6]. Head-mounted eye trackers (often also called mobile eye trackers) are glasses with an integrated infrared source and video camera that the user wears on the head [6,17,20]. Head-mounted eye trackers also provide data streams of the point of gaze in a 3D environment (x-, y-, and z-coordinates) and pupil size for both the left and right eyes. In recent years, eye-tracking technology has advanced significantly, especially in terms of robustness, allowing it to move from research laboratories to real-world applications [6]. Eye-tracking applications can be distinguished along various dimensions. While Duchowski [21] separated between interactive (eye-based interactions) and diagnostic (visual attention analysis) applications of eye tracking, Majaranta and Bulling [17] distinguished eye-tracking applications into four areas across the dimensions of real-time versus offline analysis: eye-based interactions, attentive user interface, eye-based user modeling, and diagnostic applications. According to Majaranta and Bulling [17], eye-based interactions focus on using eye movements as explicit input for real-time interactions with a user interface, while attentive user interfaces use eye-tracking data as an implicit input to support the attention management of the user. The focus of this review is on eye-based user modeling, which relies on eye-tracking data to understand, e.g., the user’s cognitive and affective processes, traits, behavior, or intentions and develop models for their recognition. Diagnostic applications refer to the offline analysis of eye movement data to gain insights into visual and attentional processes.

Eye movement data analysis extracts so-called fixations and saccades from raw gaze point data [6,22]. Fixations are the short pauses of gaze on a specific area of interest and typically have a duration of at least 50–300 ms [22]. Saccades are the rapid jumps between two consecutive fixations and have a duration of 10 to 100 ms [6]. Several algorithms exist to detect fixations and saccades out of raw eye movement data. From fixations and saccades, several metrics can be derived. Low-level metrics are based on the fixation duration, rate, and count or the saccade duration, length, acceleration, rate, and count, as well as their statistical features, such as the mean, median, minimum, maximum, skew, or kurtosis [6,23]. High-level metrics make use of so-called areas of interests (AOIs), i.e., particular areas of a UI or the environment that are of high importance, and focus on transitions between AOIs or fixation- or saccade-based metrics computed separately for each AOI [6,23].

A fundamental assumption in eye tracking is the eye–mind hypothesis by Just and Carpenter [24]. The eye–mind hypothesis states that what a person fixates on is also actively cognitively processed [24]. Research also showed a relationship between the pupil size and cognitive workload, as well as the arousal level [25,26]. This is also the reason why eye trackers are often used to study cognitive and affective processes or personality traits [11,12]. These insights into these user states and traits can be leveraged to design gaze-adaptive systems. Adaptive systems research has a long history dating back to Pope et al. [13], who suggested biocybernetic systems that rely on EEG sensors to assess operator engagement and adapt the level of automation depending on the engagement. The terms of neuroadaptive systems [1,15], physiological computing systems [4,14], physioadaptive systems [27], and biosignal adaptive systems [3] then evolved out of it. All these systems follow the same loop structure with three stages: (1) the collection of biosignals from users by sensors, (2) the recognition of user traits and states, and (3) system adaptation. In the first stage, the signals are recorded by sensors [1]. Moreover, this stage includes three components, namely, signal acquisition, signal processing, and signal storage [27]. Typically, these signals are acquired from sensors, like EEG, ECG, EDA/GSR, or eye tracking. In the second stage, the collected sensor data are analyzed with the goal of recognizing user traits and states [4]. Often, the collected sensor data are combined with self-reported data from users, e.g., collected through surveys. This process typically involves feature engineering and an analytics engine that applies supervised machine learning techniques [27]. In the final stage, the system adaptively responds based on the recognized user traits and states [14,15]. The goal of the system adaptation is either to trigger a desirable response or to maintain a desirable state [27]. Our SLR specifically focused on the first two stages. In doing so, we lay the foundations for the development of gaze-adaptive systems that use eye-tracking data as the biosignal.

## 3. Materials and Methods

To answer the research question that guides this article, we first conducted a systematic literature review. This provided a set of relevant papers on the eye-based recognition of user traits and states. In a second step, we developed a conceptualization of eye-based user recognition in the form of a framework. Based on the identified literature and the framework, we highlight research gaps and outline future research directions.

### 3.1. Systematic Literature Review

This literature review followed the guidelines established by Page et al. [28] and Kitchenham and Charters [29]. We structured the literature review process along the three phases of plan, conduct, and report. The review was not registered and a protocol was not prepared. As a first step in the planning phase, we developed the search strategy for this literature review. Starting with an exploratory search on Google Scholar, we identified the first relevant literature. After reviewing the literature found and its keywords, we iteratively developed the search string several times. The final search string consisted of four parts to capture a broad range of relevant studies that investigated user states and traits. The first part ensured that only studies that used eye, gaze, or pupil data were included. The second part focused on capturing studies that investigated the prediction of cognitive, mental, affective, emotional, physiological, psychological, or personality user constructs. The third part ensured that only studies that investigated user states, traits, or characteristics were included. The fourth part ensured that only studies were included that examined the recognition, detection, classification, modeling, and prediction of user states and traits. Therefore, after applying Boolean operators and wildcards, the following search string was developed:

(eye * OR gaze* OR pupil*)
AND (cognit* OR mental OR affect* OR emotion* OR physiolog* OR psycholog* OR personality)
AND (state OR trait OR characteristic*)
AND (recogni* OR detect* OR classif* OR model* OR predict*).

As a next step, we selected the ACM Digital Library and IEEE Explore databases for this review, as these databases are well established and considered as reliable sources for conducting literature reviews by scholars. The databases were searched on 12 November 2024. Moreover, we developed selection criteria for including studies in this review, which are presented in Table 1. We decided to exclude studies that focused on the recognition of diseases and disorders, as this requires specific medical knowledge and can have a serious impact on people’s lives if misclassified. In order to obtain a holistic overview, we decided not to limit the results to a specific type of eye-tracking data, time period, publication type, or publication outlet.

**Figure 1:**
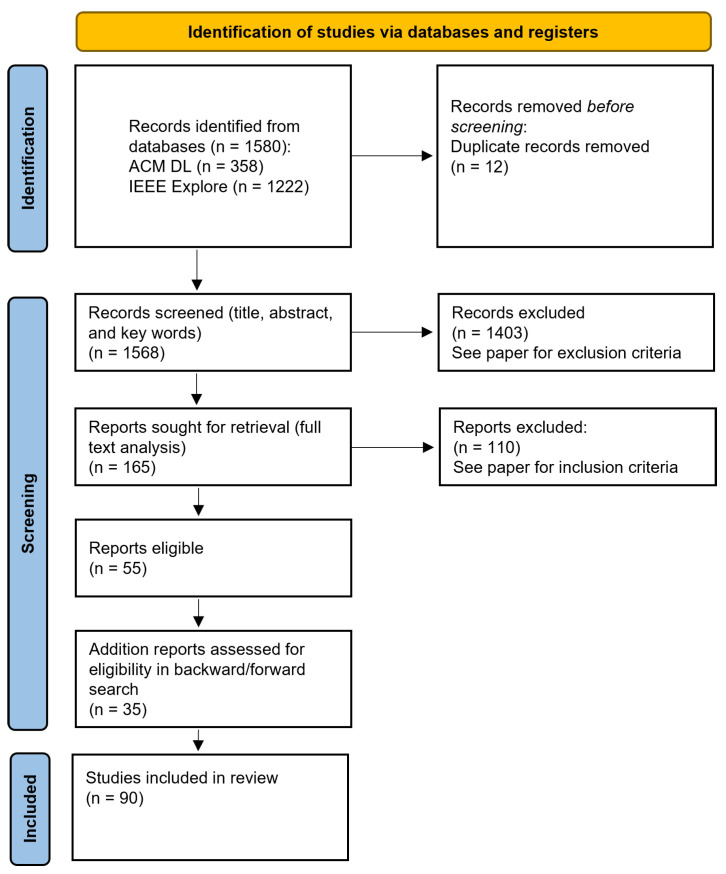
Filtering process following Page et al. [30] to select papers included for this review.

This was followed by the phase of the literature search. The previously defined search string with a filter for the abstract and title was executed on the selected databases and 1568 articles were identified, as shown in Figure 1. To identify relevant publications, we followed a single screening approach and all records were assessed by one reviewer. First, the titles, abstracts, and keywords were scanned before reviewing the full text of the publications. From this set of relevant publications, a forward and backward search was performed following the same selection criteria. The final set of publications was considered in this literature review. The result of the literature search phase was a comprehensive set of 90 papers that employed machine learning for the eye-based recognition of user traits and states.

### 3.2. Framework Creation

After identifying the relevant literature, the publications were analyzed by reading and extracting all the relevant information to provide an answer to our research question. To structure the analysis process, we developed a framework skeleton that represented all the relevant dimensions of eye-based recognition. We followed a conceptual-to-empirical and empirical-to-conceptual development approach, as suggested by Nickerson et al. [31]. To derive a first version of the conceptual framework, we first leveraged the PACT framework, as presented in Benyon [32], and gathered the previously introduced biosignal-adaptive systems concept from Hettinger et al. [1], Schultz and Maedche [3], Pope et al. [13], Allanson and Fairclough [14], Loewe and Nadj [27], and Riedl and Léger [15] to follow a top-down conceptual-to-empirical approach. By extracting these dimensions, we derived four dimensions: task, context, technology and data processing, and recognition target. In the second step, an empirical-to-conceptual approach was conducted following Nickerson et al. [31]. To derive the subcategories of each dimension and the corresponding codes, we used an inductive coding procedure based on Wolfswinkel et al. [33]. To minimize the bias, some papers were coded jointly by two reviewers, and the subcategories and codes were iteratively refined by them. Once a sufficient level of abstraction of the conceptual framework and codes was reached, one reviewer coded the papers in the final set of this review accordingly and recorded the results in a framework presented in Section 4 and a concept matrix presented in Appendix A.2.

## 4. Results

In this section, we describe the results of the literature review and analyze the identified papers in detail. Moreover, we present the framework for the eye-based user recognition of user traits and states based on the discovered literature. Finally, we provide research gaps and directions for future research.

### 4.1. Descriptive Results

**Figure 2:**
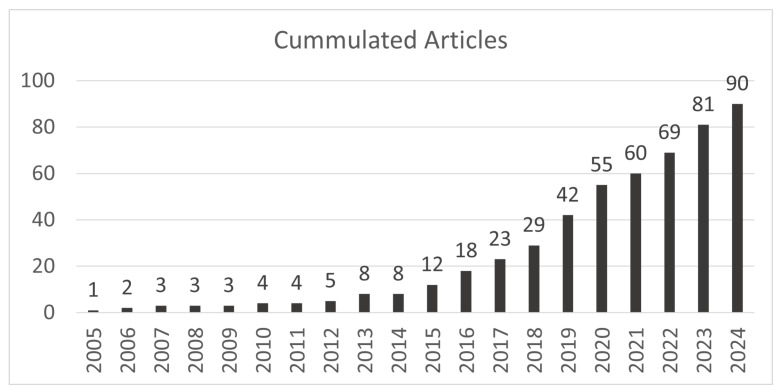
Number of articles about eye-based recognition of user traits and states accumulated per year.

As described in the Materials and Methods Section, the developed search string was applied to the relevant databases and resulted in 1568 hits. We applied the previously described inclusion criteria to this set and excluded 1403 papers based on the title and abstract. The remaining 165 publications were reviewed in detail and 55 papers remained after the full-text analysis. Most of the excluded studies either did not apply machine learning algorithms on the collected eye movement data to recognize user traits and states or focused on the eye-based recognition of activities or diseases. Finally, we performed a forward and backward search and identified an additional 35 publications. At the end of this process, the final set consisted of 90 relevant publications. A complete list of all identified papers is attached in Appendix A.1. As shown in Figure 2, analyzing the descriptive data of the publications highlighted that about ten years ago, the application of machine learning algorithms on eye-based data to recognize user traits and states emerged. Since then, there has been a steady increase in publications about eye-based user trait and state recognition. The sample sizes of the studies were quite different, with an average of M = 45.6 (SD = 46.6).

### 4.2. Framework

**Figure 3:**
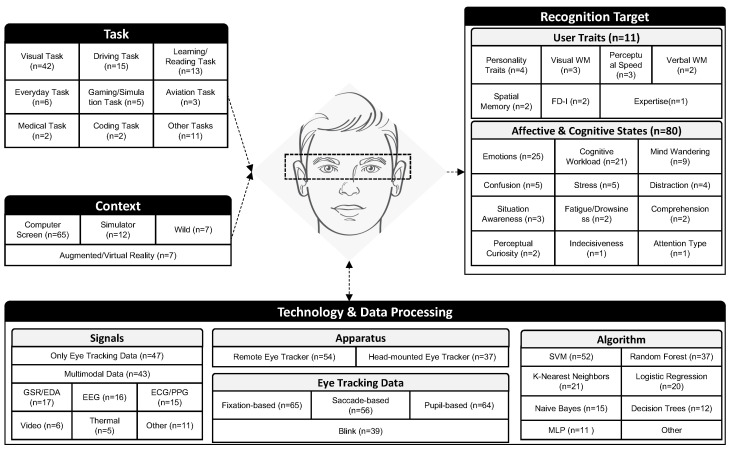
Framework of eye-based recognition of user traits and states.

Following the conceptualization process described in the previous section, a framework for the eye-based recognition of user traits and states was iteratively developed to reflect the state-of-the-art research landscape. The framework covered four dimensions: task, context, technology and data processing, and recognition target (traits and states). Figure 3 shows the (sub)dimensions, including their codes. For each code, the figure shows the number of papers in the final set that covered the respective analysis results (indicated by the number in brackets). In the following sections, we describe each (sub)dimension in detail.

#### 4.2.1. Dimensions: Task and Context

The task dimension describes the experimental task that participants had to accomplish during the studies. As it can be seen, a variety of tasks were used in the experimental studies during which eye-tracking data were recorded to recognize the user states and traits. In total, nine different task categories were identified. The most common tasks were visual tasks, in which the study participants, for example, had to work on a visual task (*n* = 42) such as exploring a visual stimulus (e.g., [10,34]), performing the n-back task (e.g., [35,36]), or the Stroop test (e.g., [37,38]). The to-be-explored visual stimulus was provided either in the form of an image (e.g., [39]), a video (e.g., [40]), or an information visualization (e.g., [41]). The second-most common tasks were driving tasks (*n* = 15) in a simulator (e.g., [42]) or in virtual reality (e.g., [43]). Other commonly studied tasks were learning and reading tasks (*n* = 13) (e.g., [9,44]), everyday tasks (*n* = 6) (e.g., [45]), gaming or simulation tasks (*n* = 5) (e.g., [7,8]), aviation-related tasks (*n* = 3) (e.g., [46]), medical tasks (*n* = 3) (e.g., [47,48]), or coding-related tasks (*n* = 2) ([49,50]). Furthermore, a few studies (*n* = 11) investigated tasks that did not fit in to the abovementioned task categories.

In terms of the context, most studies were conducted on computer screens (*n* = 65) in a controlled environment. Twelve studies were conducted in simulators, such as a driving simulator, and seven studies were conducted in the wild, such as during everyday tasks. Furthermore, seven studies investigated the recognition of user traits or states in virtual or augmented reality.

#### 4.2.2. Dimension: Technology and Data Processing

The technology and data-processing dimension includes all technical and data-related sub-dimensions, including types of signals, apparatus, collected eye-tracking data, and applied recognition algorithms.

Signals: For the biosignals collected from participants within the studies, we distinguished between mono-modal eye-tracking data collection and a multi-modal data collection that combined eye tracking with other sensor data. Half of the articles (*n* = 47) only recorded eye-tracking data to recognize user traits and states. A multi-modal approach was followed by 43 of the analyzed publications. Here, eye tracking was coupled with a variety of other biosignal sensors, such as GSR/EDA (*n* = 17) (e.g., [51,52]), EEG (*n* = 16) (e.g., [53,54]), heart-related sensors like ECG and PPG (*n* = 15) (e.g., [55,56]), video input (*n* = 6) (e.g., [57,58]), or thermal sensors (*n* = 5) (e.g., [59,60]). In 11 of the studies, speech, audio, respiration, speech, or environment information was also used as an input. Adding another modality to eye tracking has the advantage of collecting complementary information and improving the recognition accuracy [61,62].

Apparatus: In terms of the study apparatus, 54 of the studies used a remote eye tracker, while 37 of the studies relied on a head-mounted eye-tracking device. It is interesting to note that some studies with a computer-screen-based context even relied on a head-mounted eye tracker (e.g., [8,10]). Most of the used remote eye trackers were from Tobii, while SMI eye-tracking glasses were the most used head-mounted eye trackers.

Eye-tracking data: Eye-tracking devices typically provide at least two data streams, namely, a raw gaze stream and a pupil data stream. These data streams are then aggregated into fixations, saccades, pupil size, and blinks before low-level and high-level eye-tracking metrics are computed from them. These eye-tracking metrics serve as the input features for the algorithms to recognize user states and traits. Most publications (*n* = 65) relied on fixation-based features, like the number of fixations and average fixation duration. Furthermore, saccade-based features (e.g., number of saccades, average saccade length) were leveraged in 56 studies and pupil-based metrics (e.g., pupil diameter) in 64 of the studies. Blink-related metrics served as input features in 39 studies. Typically, further statistical features, such as the minimum, maximum, mean, median, standard deviation, skew, and kurtosis, were computed for all the features (e.g., [63]).

Algorithm: This literature review focused only on articles that leveraged more advanced analytical algorithms based on machine learning and deep learning approaches to recognize or predict the user trait or state. The most commonly used machine learning algorithms were Support Vector Machines (SVMs) (*n* = 52), followed by Random Forests (*n* = 37). Moreover, k-Nearest Neighbors (*n* = 21), Logistic Regression (*n* = 20), Naive Bayes (*n* = 15), Decision Trees (*n* = 12), and Multilayer Perceptrons (*n* = 11) were frequently applied algorithms in the final set of articles. The “other category” contained a wide range of different algorithms that were tested regarding its prediction quality in recognizing user states and traits, and an extensive list of algorithms can be found in Appendix A.2. Some studies also applied multiple algorithms to the collected eye-tracking data and compared them (e.g., [40]).

#### 4.2.3. Dimension: Recognition Targets

The recognition target dimension describes the user’s perspective. Specifically, it summarizes the recognized target constructs in terms of the traits and states using advanced analytical algorithms described in the previous section. This dimension is divided into two sub-dimensions of user traits and states. Overall, several papers investigated several constructs of these two sub-dimensions in parallel, like [64].

User traits: Predicting user traits through eye-tracking data was performed by 11 of all the identified publications. Typical user traits predicted through eye-tracking data were personality traits (*n* = 4). Research showed that affective personality traits (e.g., tactics, views, morality traits of the Dark Triad) were predicted through eye-tracking data more accurately than cognitive (e.g., openness of the HEXACO traits) or behavioral (e.g., conscientiousness of the HEXACO traits) personality traits [10,40]. Visual working memory capacity (WMC) and perceptual speed were investigated in three studies. Spatial memory and verbal WMCs were studied in two studies each. Verbal and visual WMCs and perceptual speed have important impacts on information processing, and detecting these cognitive abilities can enable user-adaptive information visualization [65,66]. Furthermore, two of the studies used eye-tracking data to infer cognitive styles, such as field dependence–independence during task performance ([67,68]). Raptis et al. [67] uncovered that participants with different cognitive styles could be especially distinguished based on gaze entropy, fixation duration, and count. Only one study predicted expertise based on eye data (e.g., [47]).

Affective and cognitive states: Eye-tracking data were also successfully applied to recognize specific user states by 80 of the studies. The most common state studied in more than every third study of this final set were emotions (*n* = 25). Emotions were often inferred in a multi-modal setup, together with EEG (e.g., [42,55,62]). Moreover, work not only focused on inferring the dimensions of affect, such as arousal and valence [69,70], but also on the specific types of emotions [62,71] with the help of eye-tracking data. Another user state often investigated in our publication set was cognitive workload (*n* = 21). Specifically, research established a relationship between the pupil size and the experienced cognitive workload of the study participants [35]. Furthermore, eye-tracking metrics, such as fixation-, saccade-, and blink-based metrics are also leverage for cognitive workload estimation [60,72]. The third-most frequently investigated cognitive state was mind wandering (*n* = 9), which was very often predicted during reading and learning tasks (e.g., [9,16,51]). Five of the studies used eye-tracking technology to examine confusion, which is studied in a visual task context (e.g., [73,74,75]), medical tasks (e.g., [48]), or physical tasks (e.g., [76]). Stress, which can include both cognitive and affective aspects, was examined in five studies (e.g., [38,50]). Distraction was another investigated cognitive state, where four of the studies studied it, and all of these distraction studies used driving tasks [77,78,79,80]. Other cognitive states recognized through eye-tracking data using advanced algorithms were situation awareness (*n* = 3) ([37,81,82]) and fatigue (*n* = 2) [83,84]. Moreover, two studies investigated comprehension [16,44] and perceptual curiosity [64,85] prediction, while one study investigated the recognition of indecisiveness [86] and another one the attention type [59] based on eye-tracking data.

## 5. Discussion

In this section, we propose several possible future research directions for the eye-based recognition of user traits and states considering the analysis within the framework presented in the previous section. Each dimension of the framework is discussed separately below. A condensed summary is presented in Table 2.

### 5.1. Task

A deep dive into the tasks studied in the collected papers highlights their diversity. We hypothesized that this diversity can be observed because users’ eye movements are highly task-specific and central to task performance. In addition, most tasks were studied in controlled environments, such as laboratories or highly controlled desktop environments. However, many tasks take place in semi- or highly uncontrolled environments, such as at work, school, or university. For example, working in a workshop or on an assembly line as an industrial example, teachers and professors imparting knowledge to students or supervising students at school, and white-collar workers videoconferencing or working with GenAI have not been the focus of research on the eye-based recognition of user states and traits. Therefore, we propose to further increase the diversity of tasks investigated to include tasks in semi- or uncontrolled environments. Furthermore, as unique and individual as eye movements are in different tasks, it is important to investigate different eye movement features and algorithms that work across tasks. Ensuring that models trained on one task also work on another task would support the generalizability of eye-based user models across tasks and help eye tracking to support more everyday tasks.

### 5.2. Context

An examination of the identified contexts shows that the previous research was mainly limited to the context in which users work with computer screens. However, users may perform their tasks on smaller screens, such as smartphones, or even larger screens, and thus, experience different affective and cognitive states. Focusing only on highly controlled contexts and environments also limits the ecological validity of the user state and trait models developed, as they may behave differently on data collected outside of controlled conditions. For example, bright sunlight, different poses, or larger screens or distances to screens can pose unique challenges to eye-based user models. Furthermore, gaze-adaptive systems can be used in specific spatial contexts, such as offices, production lines, and public displays with varying environmental conditions. Therefore, varying conditions and environments should be the focus of future research. In addition, new virtual environments, such as mixed reality, augmented reality, or virtual reality, should be further explored as eye tracking becomes one of the main interaction methods of these devices, as seen in the Apple Vision Pro and Meta Quest Pro. Overall, there is a need to further diversify the context in which eye-based recognition is tested.

### 5.3. Technology and Data Processing

Regarding the signal sub-dimension, the current result shows that there was limited research on using eye tracking in combination with other sensor technologies to perform recognition. However, the use of multi-modal data, such as EEG for brain activity or ECG for heart rate, is known to increase the accuracy of models [80,87]. By adding another perspective of the bodily response to changing user states, it can help to generalize the user state independent of the specific task and context. Furthermore, interaction information, such as mouse, keyboard, or application information, has not been well utilized [73]. Adding this information to multi-modal models would allow for more information about the task to be integrated into the models, potentially improving model performance. Therefore, future research should focus on the multi-modal aspect. Furthermore, it is necessary to compare different possible combinations of biosignals and interaction data to find the best one for the prediction results.

In terms of model development and evaluation, we have seen that almost every paper follows a different approach. These approaches range from train–test splits, train–test–validation splits, cross-validation, leave-one-participant/activity-out cross-validation of different metrics for evaluating models, such as accuracy; precision; recall; f1-score; AUC; or visualizations, such as the ROC curve. This makes it difficult to compare models across studies and is the reason why we do not report the model performance information in this paper. However, there is a need to define standards and best practices for reporting model evaluation results to further advance this field of research. Therefore, we encourage researchers to publish the underlying datasets and corresponding model development pipelines as open source to establish best practices and ensure correct model evaluation. Furthermore, by publishing this information, other researchers can build on this knowledge and further improve eye-based user models or test new approaches on old datasets. This would further support the development of generalizable user state and feature models.

### 5.4. Recognition Target

In general, the results showed that eye tracking can be used to detect a variety of user traits and states. Humans are complex, and psychology has examined a variety of constructs related to user traits and states that could potentially be predicted by eye-tracking data. Therefore, there is great potential for the further exploration of user traits and states. For affective–cognitive states, multi-modal approaches should be pursued. Also, previous studies focused only on the individual level, and the team level is not well explored. In collaborative scenarios, the recognition of individual and team-level affective and cognitive user states and characteristics, such as shared attention, collaborative workload, group stress, emotional competence, and social cohesion, are considered interesting. Furthermore, machine learning approaches have only been used for retrospective and not for ad hoc real-time recognition. Real-time recognition can support the simultaneous evaluation of multiple recognition targets to identify dependencies and correlations between them. For example, the system can identify user traits that influence or are influenced by certain user states. Therefore, we argue that the real-time recognition of user traits and states is a research gap and a promising avenue.

### 5.5. General Suggestions

The ability to recognize user traits and states using eye-tracking technology and machine learning approaches is the foundation for designing gaze-adaptive systems. There are many open research challenges in this exciting field. A major challenge will be the realization of the real-time recognition of user traits and states in order to enable so-called gaze-adaptive systems. Gaze-adaptive systems are a new class of adaptive systems that leverage eye-tracking data, more specifically gaze data, as the biosignal. Besides the goal of designing technology for productivity and maximizing usage time, there is a call for designing for well-being in HCI [88,89,90]. Since many user traits and states are related to well-being, there is a potential for researching gaze-adaptive systems to increase well-being. Furthermore, many different types of adaption are possible when building gaze-adaptive systems. Research should be conducted on how to design gaze-adaptive systems using various adaption strategies, such as changing the layout of the UI, adjusting the task difficulty, or providing supportive feedback to better meet the user’s needs. Looking at the study design of eye-based user model development studies, many studies recorded a few user state or trait labels per participant and the majority of studies had less than 50 participants. To advance eye-based user modeling and develop generalizable models, it is important to collect data from more participants and more labels per participant. Thus, we argue that there is a need for longitudinal and large-scale studies on eye-based user modeling. Another important aspect to consider is the data privacy of eye-tracking data, as they are considered particularly worthy of protection according to laws in European countries. Therefore, research on the anonymization of eye-tracking data and user trait and state modeling pipelines working with anonymized data is necessary to meet the requirements of the GDPR.

### 5.6. Limitations

Although rigor and relevance were emphasized in this study, we recognize that this literature review had some limitations. First, due to the selected databases and the search string developed, there was a risk of missing relevant articles. A methodological limitation of this review was that we followed a single-screen approach, which might have introduced biases in the results. Furthermore, we are well aware that the defined selection criteria may introduce a bias in the extracted results and influence the identified future research directions. To counteract potential biases, we followed the well-established guidelines from Page et al. [28] and Kitchenham and Charters [29]. Moreover, we explicitly described the procedure of this literature review. Second, a high number of the articles that we identified in this review were found through a forward and backward search. This indicates that our search string might have some shortcomings. Third, we developed a conceptual framework by examining the collected papers, which to some extent reflects the opinion of the authors. Therefore, we followed the approach of Nickerson et al. [31], applied an iterative procedure, and bilaterally developed the (sub)dimensions and codes of the conceptual framework. Fourth, we excluded papers that applied eye tracking to disease detection, as this is a medical topic that requires specific knowledge and tools. This could have had an impact on the holistic view that we aimed for in this literature review, as the approaches used for recognition in medical-related user traits and states were not covered in this review.

## 6. Conclusions

Eye tracking is already widely used in research to model and recognize user traits and states. However, previous studies were dispersed across multiple disciplines, faced limitations in ecological validity, and lacked the comprehensive overview needed to develop robust and generalizable eye-based models for user trait and state recognition. In this review, we systematically structured and analyzed the existing literature. We propose a conceptual framework and conduct an in-depth analysis of 90 relevant papers based on its dimensions. This structured overview provides researchers and practitioners with a clear understanding of the research landscape in eye-based user state and trait modeling. Additionally, it allows them to identify the best practices and reference state-of-the-art approaches in their respective fields. Furthermore, we outlined future research directions based on the framework’s (sub-)dimensions, helping researchers and practitioners formulate new research questions and expand the body of knowledge in this domain. Ultimately, we believe that this literature review serves as a foundation for developing gaze-adaptive systems that leverage eye-tracking technology to enhance user interaction and adaptation.

## Figures and Tables

**Table 1:** Inclusion criteria.

No	Inclusion Criterion Description
1	Applied a high-quality video-based eye-tracking device to collect eye data.
2	Investigated a user trait or state with eye data.
3	Leveraged eye-tracking data collected within an experimental study.
4	Applied an advanced algorithm that leveraged a machine learning or deep learning approach to recognize a user state or trait.
5	Publication should be available in English.

**Table 2:** Future research directions of eye-based user state and trait recognition.

Dimension	Suggestions
Task	Diversify studied tasks to generalize eye-based user trait and state models more independently from the specific task and to support more everyday life tasks.
Context	Diversify investigated contexts, e.g., smaller and bigger screen sizes, wild/natural environment of users (work, home, university, school), or MR/AR/VR contexts.
Technology and data processing	Follow-up multi-modal approaches for trait and state recognition (e.g., eye tracking + ECG, EEG, webcam, speech, interaction data). Build up best practices by sharing datasets and machine learning pipelines for eye-based user state and trait model development.
Recognition target	Eye-based recognition of team/group-level constructs. Simultaneous and real-time recognition of user states and traits.
General suggestions	Build gaze-adaptive systems that leverage real-time eye-based recognition. Design and evaluate different adaptation types for gaze-adaptive systems. Conduct longitudinal and large-scale studies to strengthen the generalizability of eye-based user models. Perform research on privacy-aware eye-based user state and trait recognition and gaze-adaptive systems.

## Data Availability

Data are contained within this article.

## Abbreviations

The following abbreviations are used in this manuscript:

AOI	Area of interest
AUC	Area under curve
EEG	Electroencephalogram
ECG	Electrocardiogram
EDA	Electrodermal activity
GSR	Galvanic skin response
HCI	Human–computer interaction
ML	Machine learning
ROC	Receiver operating characteristic
SLR	Systematic literature review
SVM	Support Vector Machine
UI	User interface
WMC	Working memory capacity

## Appendix A

### Appendix A.1. List of All Identified Publications

Abbad-Andaloussi et al. [49] Abdelrahman et al. [59] Abdurrahman et al. [56] Alcañiz et al. [91] Alhargan et al. [8] Alhargan et al. [69] Appel et al. [35] Appel et al. [92] Appel et al. [7] Aracena et al. [39] Babiker et al. [93] Bao et al. [57] Bao et al. [94] Barral et al. [65] Behroozi and Parnin [50] Berkovsky et al. [10] Bixler and D’Mello [95] Bozkir et al. [43] Bühler et al. [51] Castner et al. [47] Chakraborty et al. [36] Chen et al. [46] Chen et al. [96] Chen and Epps [97] Conati et al. [41] Conati et al. [98] Dumitriu et al. [99] Fenoglio et al. [100] Gong et al. [101] Gong et al. [34] Hoppe et al. [64] Hoppe et al. [85] Horng and Lin [83] Hosp et al. [48] Hutt et al. [102] Hutt et al. [103] Hutt et al. [9] Jiménez-Guarneros and Fuentes-Pineda [54] Jyotsna et al. [104] Katsini et al. [68] Kim et al. [81] Ktistakis et al. [72] Kwok et al. [105] Lallé et al. [73] Zhao et al. [106]	Li et al. [84] Li et al. [37] Liang et al. [77] Liu et al. [78] Liu et al. [107] Liu et al. [108] Lobo et al. [109] Lu et al. [61] Lufimpu-Luviya et al. [86] Luong and Holz [110] Luong et al. [111] Ma et al. [112] Mills et al. [113] Mills et al. [63] Misra et al. [79] Miyaji et al. [80] Mou et al. [42] Oppelt et al. [60] Raptis et al. [67] Reich et al. [44] Ren et al. [114] Ren et al. [52] Salima et al. [45] Salminen et al. [74] Sims and Conati [75] Soleymani et al. [53] Steichen et al. [66] Stiber et al. [76] Tabbaa et al. [55] Taib et al. [40] Tao and Lu [115] Tarnowski et al. [70] Wang et al. [116] Wu et al. [117] Qi et al. [16] Xing et al. [58] Yang et al. [118] Zhai et al. [38] Zhai and Barreto [119] Zhang et al. [120] Li et al. [121] Zheng et al. [62] Zheng et al. [71] Zhong and Hou [122] Zhou et al. [82]
